# Role of HO/CO in the Control of Peripheral Circulation in Humans

**DOI:** 10.1155/2012/236180

**Published:** 2012-03-04

**Authors:** David Sacerdoti, Despina Mania, Paola Pesce, Silvia Gaiani, Angelo Gatta, Massimo Bolognesi

**Affiliations:** Department of Clinical and Experimental Medicine, Clinica Medica 5, University of Padova, Via Giustiniani 2, 35100 Padova, Italy

## Abstract

Experimental studies show that the heme oxygenase/carbon monoxide system (HO/CO) plays an important role in the homeostasis of circulation and in the pathophysiology of hypertension. No data are available on its role in the control of peripheral circulation in humans. We evaluated the effects of inhibition of HO with stannous mesoporphyrin IX (SnMP) (200 *μ*M) locally administered by iontophoresis, on human skin blood flow, evaluated by laser-Doppler flowmetry, in the presence and absence of nitric oxide synthase (NOS) inhibition with L-NG-Nitroarginine methyl ester (L-NAME) (100 *μ*M). We also evaluated the effect of HO inhibition on vasodilatation induced by acetylcholine (ACh) and vasoconstriction caused by noradrenaline (NA). SnMP and L-NAME caused a similar 20–25% decrease in skin flow. After nitric oxide (NO) inhibition with L-NAME, HO inhibition with SnMP caused a further 20% decrease in skin perfusion. SnMP decreased vasodilatation induced by ACh by about 70%, while it did not affect vasoconstriction to NA. In conclusion, HO/CO participates in the control of peripheral circulation, independently from NO, and is involved in vasodilatation to ACh.

## 1. Introduction

Carbon monoxide (CO) is physiologically produced in the arterial wall by the action of heme oxygenase (HO) on heme. HO exists in two isoforms: the inducible HO-1, and the constitutive HO-2 [1, 2].

CO, generated in endothelial and smooth muscle layers of blood vessels by HO, modulates vascular tone, by inducing relaxation of vascular smooth muscle cells. Smooth muscle cell relaxation is obtained by stimulating soluble guanylyl cyclase (sGC) and by opening large-conductance calcium-activated K^+^ channels (BK-Ca) [3]. CO acts on smooth muscle cells through mechanisms that are also involved in the action of other vasoactive substances, such as nitric oxide (NO) and endothelium-derived hyperpolarizing factor (EDHF) [4, 5]. CO also inhibits the cytochrome P450-dependent monooxygenase system, with a decrease in 20-hydroxyeicosatetraenoic acid (20-HETE), which sustains contractile tone by inhibiting potassium channels [4]. In normal rats, inhibition of HO with chromium mesoporphyrin impairs ACh-induced vasorelaxation only when cyclooxygenase, NO synthase, and sGC are all inhibited, while it has no effect in baseline condition [3]. Nonetheless, HO inhibition increases peripheral vascular resistance and blood pressure in experimental animals, but no data are available in humans [2, 4].

Aim of this study was to evaluate whether the HO/CO system participates in the control of peripheral circulation in humans. We have chosen skin circulation as it is involved in the control of peripheral resistance and can be estimated by the laser-Doppler technique, which allows to evaluate not only skin flow but also the local effects of vasoactive substances and inhibitors administered locally by iontophoresis.

## 2. Patients and Methods

The study was performed in 20 normal volunteers, age 24–50 yr, 12 males and 8 females. An informed consent was obtained from each subject, and the study was approved by the institutional local ethical committee.

After at least a 4-hour fast and 10 min lying on a bed at 21–24 C, a laser Doppler probe was applied on the dorsal face of the 2nd phalanx of the 3rd finger and skin flow, expressed as arbitrary units (PFUs), was measured for 5 minutes. The average value was considered as the basal flow.

After baseline measurement, the HO inhibitor stannous mesoporphyrin IX (SnMP) (200 *μ*M) (Frontiers Scientific, Logan, UT, USA) was applied by iontophoresis (four 15-second infusions at 0.06 mA) in the same skin area and its effects were evaluated, while another probe was measuring flow in another finger of the other hand in order to exclude changes of flow not due to the inhibitor. The effect of the NOS inhibitor, L-NG-Nitroarginine methyl ester (L-NAME) (100 *μ*M) (Sigma Chemicals, St. Louis, MO, USA), similarly applied by iontophoresis, was then evaluated on another finger. The HO inhibitor SnMP (200 *μ*M) was then applied together with L-NAME and the effect of the two inhibitors was measured.

The effects of noradrenaline (NA) (10 mMol) (Sigma Chemicals, St. Louis, MO, USA) (four 15-second infusions at 0.06 mA) and of the endothelium-dependent vasodilator, acetylcholine (ACh) (11 mMol) (Sigma Chemicals, St. Louis, MO, USA) (seven 15-second infusions at 0.06 mA, with 45-sec intervals), applied by iontophoresis, were evaluated in different subjects, in the presence or absence of HO inhibition with SnMP.

Laser Doppler flowmetry gives a semiquantitative assessment of microvascular blood perfusion, reflecting perfusion of capillaries, arterioles, venules, and dermal vascular plexa [6]. Measurements were done using a PeriFlux laser Doppler flowmeter (LDF) (Periflux system 5000, Perimed, Jarfalla/Stockholm, Sweden). Iontophoresis allows transdermal delivery of polar compounds by means of a small electrical current. The delivery is done in the same area where blood perfusion is measured, allowing the assessment of microvascular reactivity when blood perfusion is measured simultaneously. 

### 2.1. Statistical Analysis

Results were shown as mean ± SD. Differences among groups were analyzed by Anova and unpaired Student's *t*-test. Statistical significance was set at *P* < 0.05.

## 3. Results

**Figure 1:**
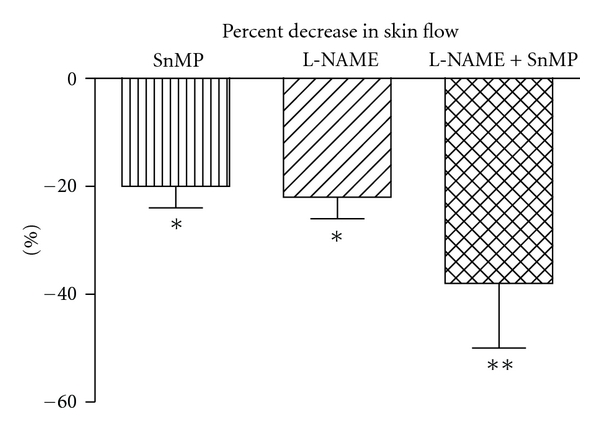
Effect of heme oxygenase inhibition with stannous mesoporphyrin IX (SnMP) (200 *μ*M), of nitric oxide synthase inhibition with L-NG-Nitroarginine methyl ester (L-NAME) (100 *μ*M), and of both, on skin blood flow measured by laser Doppler flowmetry. SnMP and L-NAME were administered locally by iontophoresis. *P <0.05 versus baseline; **P <0.05 versus L-NAME.

**Figure 2:**
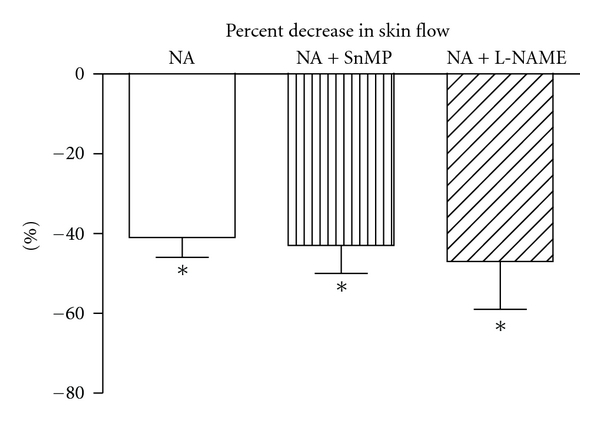
Effect of heme oxygenase inhibition with stannous mesoporphyrin IX (SnMP) (200 *μ*M) and nitric oxide synthase inhibition with L-NG-Nitroarginine methyl ester (L-NAME) (100 *μ*M) on the vasoconstricting effect of noradrenaline (NA) on skin blood flow measured by laser Doppler flowmetry. NA, SnMP, and L-NAME were locally administered by iontophoresis. *P <0.05 versus baseline.

**Figure 3:**
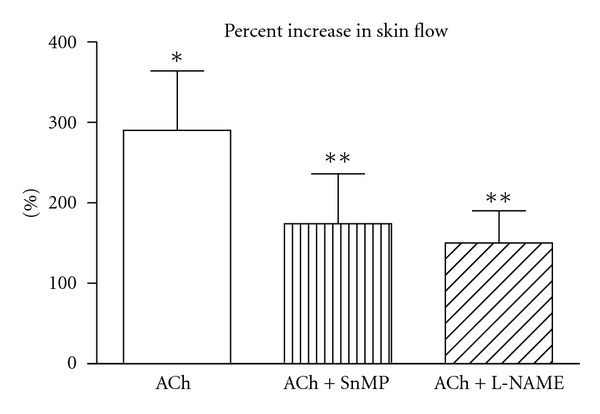
Effect of heme oxygenase inhibition with stannous mesoporphyrin IX (SnMP) (200 *μ*M) and nitric oxide synthase inhibition with L-NG-Nitroarginine methyl ester (L-NAME) (100 *μ*M) on vasodilatation induced by acethylcholine (ACh) in skin circulation evaluated by laser Doppler flowmetry. ACh, SnMP, and L-NAME were locally administered by iontophoresis. *P <0.05 versus baseline; **P <0.05 versus Ach.

Skin flow under basal condition was  23 ± 6  PFU and was stable during the whole experiment, as demonstrated on the control finger. Inhibition of HO with SnMP, administered locally by iontophoresis in the same skin area where the flow probe was positioned, caused a 20% decrease in skin flow (Figure 1). A similar decrease was shown after NOS inhibition with L-NAME (Figure 1), also given locally by iontophoresis on a different finger. Inhibition of SnMP after L-NAME almost doubled the decrease in flow (Figure 1). NA, administered locally by iontophoresis, caused a 42% reduction in skin flow (Figure 2), while ACh caused a 290% increase (Figure 3). Inhibition of HO with SnMP, or NOS with L-NAME, did not affect the response to NA (Figure 2), while both of them reduced vasodilatation to ACh by about 70% (Figure 3).

## 4. Discussion

The results of this study show for the first time a role of the HO/CO system in the control of peripheral circulation in humans.

Skin blood flow is the result of a balance between vasoconstricting and vasodilating systems. NO is considered the most important endothelium-dependent vasodilator of the skin microcirculation and is released in response to ACh and shear stress. Its synthesis from L-arginine is inhibited by L-NMMA or L-NAME. Kvandal et al. [6] have shown that intraarterial infusion of L-NMMA causes a 20% reduction in skin flow, which could be reversed by l-arginine. L-NMMA; however, it did not affect skin perfusion raised by ACh. Our results, although obtained with a different technique, confirm that inhibition of NOS causes a reduction in skin flow. Furthermore, we showed that NOS inhibition reduced the vasodilating affect of ACh by about 70%. This difference in results is probably related to the different method of administration of the NOS inhibitor, which was given locally, by iontophoresis in our subjects, and intraarterially in Kvandal's study. It is, in fact, possible that the local administration allows to obtain higher concentrations and, thus, a higher effect due to a higher inhibition of NOS, considering that the intraarterial infusion cannot be done using high doses to avoid the systemic effects. Thus, our results confirm that cutaneous vascular tone is regulated by NO.

Treatment of normotensive rats with HO inhibitors causes elevation of peripheral vascular resistance and blood pressure, suggesting that endogenous CO subserves a vasodepressor function [7]. The same conclusion was derived from reports that treatment of hypertensive rats with HO inducers or substrates reduces blood pressure via a heme oxygenase-dependent mechanism [8–11]. Also, observations that heme elicits HO-dependent vasodilation in isolated gracilis muscle arterioles and tail arteries suggested that CO of vascular origin can be a mediator of vasodilatory mechanisms.

In our normal subjects, basal skin flow was reduced by SnMP, even in the presence of NOS inhibition with L-NAME. Thus, it is conceivable that CO participates in the physiologic control of tone in the peripheral circulation and may also be involved in pathophysiology. CO-induced cell signaling has been proposed to occur via sGC activation, although CO is far less effective at activating sGC than is NO [2, 5]. CO also activates BK-Ca channels in smooth muscle cells from a variety of different vascular beds, including cerebral and tail arteries [12]. In isolated vascular smooth muscle cells, CO-induced BK-Ca channel activation is not blocked by inhibitors of sGC and is not reproduced by other products of HO-mediated heme metabolism. Thus, vascular smooth muscle cell HO-derived CO, or exogenous CO, activates arterial smooth muscle cell BK-Ca channels either directly or via interaction with channel-associated regulatory elements. Furthermore, NO and CO activate vascular smooth muscle cell BK-Ca channels via distinct mechanisms that involve effects on different channel subunits [2, 3, 5], thus explaining the additive effect of NOS and HO inhibition on skin flow.

The effect of ACh was also blunted by HO inhibition, suggesting that the NO-independent and prostacyclin-independent component of vasodilatation to ACh is mediated, at least in part, by the HO/CO system.

We have previously shown that HO inhibition in mesenteric microvessels from normal rats, pretreated with indomethacin, L-NAME, and also the cGMP inhibitor 1H-[1,2, 4]oxadiazolo[4, 3-a] quinoxalin-1-one (ODQ), causes a decrease in the vasodilating effect of ACh [3]. This effect was similar to that of iberiotoxin, a BK-Ca channels inhibitor. Thus, it is conceivable that the HO/CO system participates in the homeostasis of vascular tone by stimulating BK-Ca channels, although it is well known that other mechanisms are possible, like inhibition of 20HETE synthesis, decrease of ROS [4]. HO inhibition did not increase the vasoconstricting response to NA. This last result is similar to what shown in the rat mesenteric circulation [13]. It is possible that an increased response to NA by HO inhibition is masked by an increase in NO, prostacyclin, or other vasodilators, but there has also to be considered the possibility that the degree of vasoconstriction induced by NA was the highest which could be obtained with a vasoconstrictor (a mean of 42%) in the skin and, thus, any increase could not be demonstrated. On the contrary, when HO is induced, like in cirrhosis or after transfection with the HO-1 gene [13], the hyporeactivity to vasoconstrictors can be reversed by inhibition of HO.

The results of this study may have therapeutic implications. While inhibition of HO may be useful for treatment of vasodilatation and hyperdynamic circulation, like in sepsis or cirrhosis, in conditions of increased peripheral resistance, like hypertension, or of vasoconstriction, like angina or ischemic arteriopathy, HO induction or supplementation of CO could be employed.

In conclusion, in normal physiological conditions, in humans, the HO/CO system participates in the control of peripheral circulation, besides NO, and also mediates, in part, the endothelium-dependent vasodilatation to ACh.

## Abbreviations

CO: Carbon monoxide
HO: Heme oxygenase
NO: Nitric oxide
L-NAME: L-NG-Nitroarginine methyl ester
NOS: Nitric oxide synthase
SnMP: Stannous mesoporphyrin IX
Ach: Acetylcholine
NA: Noradrenaline.
